# The assessment of yield and quality traits of sweet potato (*Ipomoea batatas* L.) genotypes in middle Black Sea region, Turkey

**DOI:** 10.1371/journal.pone.0257703

**Published:** 2021-09-20

**Authors:** Yasin Bedrettin Karan, Özlem Gültekin Şanli

**Affiliations:** Faculty of Agriculture, Field Crops Department, Tokat Gaziosmanpaşa University, Tokat, Turkey; Harran Üniversitesi: Harran Universitesi, TURKEY

## Abstract

Sweet potato (*Ipomoea batatas* L.) cultivation in Turkey is concentrated in one province situated in Mediterranean region only, which would not fulfill the domestic needs of the country soon. Therefore, cultivation of the crop in other provinces/climatic regions should be initiated to fulfill the domestic needs. The cultivation in other provinces requires thorough assessment of yield and quality traits of target crop. Therefore, yield and quality characteristics of four sweet potato genotypes (i.e., ‘Hatay Kırmızısı’, ‘Hatay Yerlisi’, ‘Havuc’ and ‘Kalem’) were assessed in the current study in Kazova and Niksar counties of Tokat province of the country having middle Black Sea climate in field experiments during 2018 and 2019. The cuttings of the genotypes were planted in Niksar during the second fortnight of April and first week of May in Kazova. The planting density was kept 90 × 45 cm. Data relating to number of storage roots, storage root weight, storage root yield per hill and storage root yield per hectare were recorded. Furthermore, quality traits, including dry matter ratio (%), protein ratio (%) and antioxidant ratio (%) of storage roots were also determined. The highest total storage root yield was recorded for ‘Havuc’ genotype during both years and locations, followed by ‘Hatay Yerlisi’ and ‘Hatay Kırmızısı’ genotypes. Overall, storage root yield (60.06 and 62.40 tons ha^-1^ during first and second year) recorded for the experiment at Niksar was higher than the storage root yield recorded for Kazova experiment (53.50 and 52.84 tons ha^-1^ during first and second year, respectively). The highest dry matter was produced by ‘Kalem’ and ‘Hatay Yerlisi’ genotypes during both years and at both locations, followed by ‘Hatay Kırmızısı’ and ‘Havuc’ genotypes. The storage roots of the tested genotypes accumulated higher dry matter at Kazova during both years. The highest protein content was obtained from the ‘Kalem’ genotype, and the protein contents of the ‘Hatay Yerlisi’ and ‘Hatay Kırmızısı’ genotypes were close to the ‘Kalem’ genotype. The results indicated that tested genotypes can successfully be cultivated in middle Black Sea climate. Therefore, production of sweet potato can be initiated in the future to meet the domestic needs for sweet potato in the country.

## Introduction

Sweet potato (*Ipomoea batatas* L.), a member of the ivy (morning glory) family (*Conconvulaceae*), is of Latin American origin [1]. It is an important storage root crop and excellent food source for humans and quick source of glucose [2]. It is relatively drought-resistant, has high yield potential, wide adaptability and low input requirements [3,4]. The underground and above-ground organs of the plant are consumed in human and animal nutrition, respectively [5]. Sweet potato is generally produced only in the villages of İskenderun and Yayladağı districts of Hatay Province in Turkey [6]. Surveys conducted for sweet potato cultivation areas revealed it was first introduced to Hatay province with the immigrants from Crete at the beginning of the 20^th^ century [7]. Sweet potato genotypes with different skin and inner color named ‘Hatay Yerlisi’, ‘Hatay Beyazı’, ‘Havuç’, ‘Kalem’ and ‘Yellow Potato’ are currently cultivated in the province.

Sweet potato is cultivated in >100 countries with tropical and subtropical climates around the world. It ranks seventh in terms of production with 113 million tons storage roots produced annually. It is an important industrial plant for starch and alcohol raw material. It is cultivated on 9202 thousand hectares around the world with an average yield of 12200 kg ha^-1^. Sweet potato is directly consumed in human nutrition, and mostly produced in China, Vietnam, Indonesia, Philippines, Papua New Guinea, Cuba and Uganda [8]. Storage roots of sweet potatoes contain 30% dry matter, of which 70% consists of starch, 10% sugars, and 5% proteins. In addition, storage roots contain significant quantity of phytochemicals, which have beneficial health effects, while roots and leaves contain β-carotene (provitamin A), ascorbic acid (vitamin C), vitamin B complex and phenolic compounds such as anthocyanins [5,9–11]. Kosieradzka et al. [12] reported that purple and red-fleshed potatoes are rich in natural anthocyanins. The phytochemicals of sweet potato consist of high free-radical scavenging activity, which is important to prevent the chronic diseases and age-related neuronal degeneration [10].

Sweet potato is commonly grown in temperate regions; however, also adapted to mild temperate regions where temperature is above 20°C. The productivity of sweet potato is higher in low altitudes; however, it can be grown in an altitude ~2000 m, where yield is significantly lower [13,14]. Sweet potato requires heat and plenty of sun, grows better under high light intensity and frequently cultivated between 30 and 40° latitudes in both hemispheres. Average temperature should be 25°C and above for higher storage root development and yield. Ideal temperature during growing period is 30–35°C during day and 20°C at night to encourage storage root development. High night temperature decreases vegetative and storage root growth. Like high temperature, low night temperature also reduces storage root development. Growth may cease <10°C and chilling injury may occur when the plants are exposed to low temperatures. The development of sweet potato storage roots is higher under warm sunny days and moderately cool nights [15]. At least 4–6 months period without frost is required for successful sweet potato cultivation. Sweet potato is a drought-tolerant crop; however, storage root yield increases with increasing soil moisture content [1,16] due to the deep root system. Therefore, the regions with well-distributed rainfall such as temperature zones between 30 and 40° latitudes in both hemispheres or the regions with irrigation infrastructure favor the cultivation of sweet potato [1,17]. In addition, sandy-loam textured well-drained soils provides optimum environment for storage root development [18,19].

The identification of suitable production areas and genotype selection is strongly dependent on genotype-by-environment interactions [2,20,21]. Environmental/climatic conditions exert a strong impact on yield and quality traits of sweet potato owing to these interactions [22–26]. Nonetheless, this interaction has a strong influence on the performance of genotypes under varying environments and could be hurdle in the selection process [27]. Hence, assessment of yield and quality traits of different genotypes under varying environmental conditions would help their cultivation in different climatic regions [25,28–32]. However, cultivation of sweet potato in only one climatic region of Turkey deserves genotypic evaluation in other climatic regions of the country.

Therefore, this study assessed storage root yield and quality attributes of local sweet potato genotypes in middle Black Sea region of Turkey. The major objective was to determine whether cultivated genotypes in Mediterranean region of the country can adapt to middle Black Sea climate region. Determining the possibility of cultivation of the local genotypes was the second major objective of the study. It was hypothesized that local genotypes will perform well in the middle Black Sea climate and produce comparable storage root yield to that of Mediterranean region. It was further hypothesized that genotypes will differ for yield and quality attributes. The results of the study will help to expand the cultivation of sweet potato in the country.

## Materials and methods

### Materials

Cuttings of the four local sweet potato genotypes, i.e., ‘Hatay Kırmızısı’, ‘Havuc’, ‘Kalem’ and ‘Hatay Yerlisi’ cultivated in Hatay Province located in the southern region of Turkey were used as experimental material in the study. Seeds of the varieties were procured from the local market in Hatay province and used in the study. The description of different local genotypes used in the current study is summarized in Table 1.

**Table 1:** Description, origin and some morphological attributes of local sweet potato genotypes included in the study adopted from Çalışkan et al. [6].

Genotypes	Origin	Plant type	Storage-root shape	Skin color	Flesh color	Sweetness
Hatay Kırmızısı	Local	Semi-erect	Long-eliptic	Dark red	Pale yellow	Sweet
Hatay Yerlisi	Local	Semi-erect	Irregular	Dark red	Cream	Sweet
Havuç	Local	Erect	Round-eliptic	Pale yellow	Orange	Sweet
Kalem	Local	Semi-erect	Long-eliptic	Pale yellow	Cream	Sweet

### Experimental sites

The field experiments were conducted in two different districts (Niksar and Kazova) of Tokat province, Turkey. Experimental site in Niksar was situated between 40.534167°N, and 36.929167°E longitude (290 m asl) during first year (2018) and between 40.5625°N latitude and 36.913889°E longitude (281 asl) during second year (2019). Similarly, the experimental field in Kazova was situated between 40.331944°N latitude and 36.473333°E longitude (595 m asl) during first year (2018), and between 40.330278°N latitude and 36.391389°E longitude (567 m asl) during second year (2019) of the experiment.

Composite soil samples were taken from 0–30 cm depth and analyzed at soil testing Laboratory, Faculty of Agriculture, Tokat Gaziosmanpasa University. Soils of the experimental sites were clay-loam in texture and alkaline (pH 8.1 and 7.9 during first and second year, respectively). Soils had sufficient organic matter content (2.04–2.02%), high lime content (21.00–21.42%), poor in available phosphorus (P_2_O_5_; 1.04–1.31 kg ha^−1^) and rich in exchangeable potassium content (K_2_O; 13.75–12.95 kg ha^−1^) [33].

Monthly average temperature and total precipitation received at both experimental sites are given in Table 2. Tokat province is located in the inner part of the Central Black Sea region and influenced by Black Sea and Central Anatolian steppe (continental) climates. Therefore, the climate is a transition between the Black Sea climate and the Central Anatolian steppe climate.

**Table 2:** Average temperature (°C) and rainfall (mm) at Niksar and Kazova locations during the experimental years and between 1951 and 2020.

Months	Niksar						Kazova					
	Temperature (°C)			Rainfall (mm)			Temperature (°C)			Rainfall (mm)		
	2018	2019	1951–2020	2018	2019	1951–2020	2018	2019	1951–2020	2018	2019	1951–2020
**April**	15.5	12.8	13.8	9.4	57.9	35.4	14.8	11.5	12.4	4.5	63.5	53.4
**May**	19.4	19.7	18.3	77.4	48.6	68.4	18.5	19.1	16.3	59.1	49.1	59.2
**June**	22.4	23.6	21.8	45.0	57.5	68.6	22.0	23.1	19.6	41.5	26.2	38.9
**July**	25.0	22.5	23.8	5.0	30.6	14.7	24.2	21.9	22.1	7.2	16.9	11.6
**August**	24.5	23.0	24.4	6.3	50.3	24.2	23.7	22.4	22.3	3.9	52.2	8.4
**September**	21.3	19.9	21.2	3.7	19.9	21.7	20.4	19	18.8	14.2	2.9	18.0
**October**	16.8	18.2	15.6	33.1	4.1	29.4	15.7	17.4	13.8	39.6	3.7	37.0

Experimental design was randomized complete block with three replications and each plot consisted of four rows of 3.6 m. Planting density in each plot was 90 × 45 cm. Planting in both years was carried out during second fortnight of April at the Niksar and in the first week of May at Kazova. In all locations, 80 kg ha^-1^ 15N-15P_2_O_5_-15K_2_O fertilizer was applied before planting, and additional 80 kg N ha^-1^ was applied one month after planting. Mechanical weed control was carried out to prevent weed competition. Plants were irrigated by drip irrigation system at an average of ten days intervals. The crop was harvested during first fortnight of October in the Kazova, and in last week of October at Niksar.

The plants in the central two rows of each plot were removed one by one and the number of storage roots per plant, average storage root weight and the total storage root yield were determined. Dry matter ratios, protein ratios and antioxidant contents of the storage roots were calculated for both years and locations. Four g of storage root sample was weighed and placed on a watch glass in order to determine dry matter ratio. The samples were kept in an oven at 105°C for 48 hours, weighed and the dry matter ratios were calculated.

The samples were ground and powdered with the IKA A11 Basic model blender and dried in a BINDER brand oven for 48 hours at 65°C for determining protein ratio. A calibration curve was drawn using aspartic acid standard according to the sample category to be analyzed from the Thermo Cookbook before starting the analysis, samples were weighed in specified amounts and analyzed. Protein analysis was performed with the Thermo Scientific flash 2000 N-protein analyzer device.

### Antioxidant assays

#### 2,2-Diphenyl-1-picrylhydrazyl (DPPH) free radical assay

Various methods are available to evaluate the antioxidant activity of natural compounds in food or biological systems. The 1,1-diphenyl-2-picrylhydrazyl (DPPH) and 2,2-azinobis-(3-ethyl benzo thiazoline-6-sulfonate) (ABTS) are two commonly used methods for antioxidant analysis. The mechanism of both methods is similar because the absorption spectra of stable, free radicals change when the molecule is reduced by an antioxidant or a free radical species. The ABTS can dissolve in aqueous and organic solvents and react readily compared to DPPH, which normally takes several hours for the reaction to complete. The color of the DPPH test can interfere with samples containing anthocyanins, which leads to an underestimation of antioxidant activity. However, this problem does not arise in the ABTS test, especially when the absorbance value is measured at 734 nm. However, DPPH procedure can be successfully used to determine antioxidant activity of sweet potatoes [34–36].

The DPPH assay is used as a substrate to evaluate the activity of antioxidant enzymes. The DPPH^•^ free radical scavenging effect of sweet potato varieties was determined according to Blois [37]. In this method, 1.0 mL, 0.26 mM of DPPH solution was treated with sweet potatoes extract (20–80 μL) at room temperature for 30 min. Absorbance of the extract was measured at 517 nm using a spectrophotometer. Following equation (Eq 1) was employed to calculate the DPPH^•^ scavenging capacity. The DPPH kit was purchased from Merck SA (Pty) Ltd. (Sandton, Johannesburg, SA). The DPPH scavenging activity was measured according to Eq 1 given by Karan and Çadırcı [38].


$$DPPH\;scavenging\;activity\;(\%)=\frac{A1-A2}{A2}\times 100$$ (Eq 1)

Where, A_1_ is the control and A_2_ is the sample absorbance.

### Statistical analysis

The data were subjected to two-way analysis of variance (ANOVA) [39]. The data were tested for normality prior to ANOVA by Shapiro-Wilk normality test which indicated that data were normally distributed [40]. Therefore, analysis was performed on the original data. Differences among experimental years were tested by paired t test, which indicated significant differences. Therefore, data of both years were analyzed and presented separately. When ANOVA indicated significant difference between genotypes, Duncan’s multiple range test was used to measure the differences among genotypes’ means. All statistical analysis were carried out on SPSS (Version 210) software [41].

## Results and discussion

### Number of storage roots and storage root weight per plant

Average number of storage roots per plant significantly (p<0.05) differed among tested genotypes during both years at Niksar (Table 3). The highest number of storage roots per plant during both years were noted for ‘Hatay Kırmızısı’ genotype at both locations. The lowest number of storage root at all locations and years were noted for ‘Havuç’ genotype. The number of storage roots at Niksar was higher than Kazova during both years. Yield, number and weight of storage roots per plant have been used to explain sweet potato yield by Maity and Chatterjee [42], Islam et al. [19] and Afuape et al. [43].

**Table 3:** The number and weight of storage roots per plant (g) for different local sweet potato genotypes used in the study.

Genotypes	Number of storage root per plant				Weight of storage root (g per plant)			
	Niksar		Kazova		Niksar		Kazova	
	2018	2019	2018	2019	2018	2019	2018	2019
**Kalem**	6.21 b	7.20 b	5.04^NS^	5.60 b	676.67 c	942.67 b	520.00 b	706.67 b
**Hatay Kırmızısı**	9.71 a	10.67 a	8.87	9.37 a	2696.67 b	2896.00 a	2516.67 ab	2406.67 a
**Hatay Yerlisi**	8.37 ab	9.00 ab	7.10	7.70 ab	2930.00 ab	3116.67 a	2550.00 ab	2450.00 a
**Havuç**	7.82 ab	8.73 ab	6.81	7.48 ab	3426.67 a	3153.33 a	3080.00 a	2996.67 a
**CV %**	**14.99**	**13.40**	**18.00**	**16.48**	**1.43**	**1.29**	**1.55**	**1.47**
**Mean**	**8.03**	**8.90**	**6.96**	**7.54**	**2432.50**	**2527.17**	**2166.67**	**2140.00**

^NS =^ non-significant, means with different letters in a column are significantly different from each other at p<0.05.

The number of storage roots reported by Arslanoğlu and Hendekçi [44] ranged between 11 and 18 pieces per plant, which was higher than our findings. The number of storage roots per plant for 10 different sweet potato genotypes under Izmir ecological conditions varied between 4.9 and 7.7, and the highest number of storage roots was recorded for ‘Hatay Kırmızısı’ genotype (7.7), followed by ‘İstanköy’.

The tested genotypes significantly differed for weight of storage roots per plant during both locations and years. The highest storage root weight per plant at both locations was noted from ‘Havuç’ genotypes during both years. Overall, storage root weight at Niksar was higher than Kazova during both years. Storage root yield for 3 different sweet potato local genotypes was reported between 2219.4 and 2523.3 g plant^-1^ by Arslanoğlu and Hendekçi [44], whose results were compatible with the storage root weights per plant of the local genotypes used in this experiment. Yield parameters are general selection criteria for sweet potato; therefore, considered important indicators in determining the adaptability of genotypes used in this study. The weight and number of storage roots per plant and tuberous root yield have been used as adaptation criteria for other sweet potato researchers targeting storage roots production [45].

### Mean and total storage root yield (ton ha^-1^)

Storage root yield of sweet potato is related to the number of plants per unit area, number of storage root per plant, and weight of the storage roots, which may vary across environments [46]. Average storage root weight was statistically significant (P<0.01), while the difference in total storage root yield was important at 5% level of significance (Table 4). The highest storage weight was recorded for ‘Havuç’ genotype during both years, while the lowest storage root weight was observed for ‘Kalem’ genotype.

**Table 4:** Mean and total storage root weight (g) of different local sweet potato genotypes included in the current study.

Mean storage root weight (g)					Total storage root weight (ton/ha)			
	Niksar		Kazova		Niksar		Kazova	
Genotypes	2018	2019	2018	2019	2018	2019	2018	2019
**Kalem**	108.84 c	132.55 b	111.22 c	130.83 c	16.71 c	23.28 b	12.84 b	17.45 b
**Hatay Kırmızısı**	278.50 b	292.07 a	281.26 b	255.29 ab	66.58 b	71.51 a	62.14 ab	59.42 ab
**Hatay Yerlisi**	350.90 ab	324.30 a	360.46 ab	312.29 a	72.35 ab	76.95 a	62.96 ab	60.49 ab
**Havuç**	446.50 a	364.70 a	445.66 a	393.75 a	84.61 a	77.86 a	76.05 a	73.99 a
**CV %**	**4.03**	**3.62**	**3.98**	**3.85**	**9.10**	**8.21**	**9.86**	**9.37**
**Mean**	**296.19**	**278.41**	**299.65**	**273.04**	**60.06**	**62.40**	**53.50**	**52.84**

Means with different letters in a column are significantly different from each other at p<0.05.

The ‘Havuç’ genotype produced the highest storage root weight during both years and locations followed by ‘Hatay Kırmızisı’ genotype. The storage root weights reported by Yıldırım et al. [47] are in agreement with our findings. The differences in partitioning of photosynthates due to the genetic variations among sweet genotypes result in varying storage root yield [48]. Therefore, storage root yield is considered as an important indicator to determine adaptability of sweet potato genotypes. The results revealed that ‘Havuç’ genotype can be used as a good source of marketable storage roots. Similar to the high storage root yield of ‘Havuç’ genotype, Wariboko and Ogidi [18] reported higher total tuberous root yield for orange fleshed sweet potato varieties.

Storage root yield of local genotypes grown at Niksar was higher than those grown in Kazova (Table 4). Lewthwaite and Triggs [46] indicated that the number and sizes of the storage roots depend on the rooting structure and other characteristics that are highly affected by the environmental conditions.

The highest storage yield during both years was obtained from ‘Havuç’ genotype followed by ‘Hatay Yerlisi’, ‘Hatay Kırmızısı’ and ‘Kalem’ genotypes at both locations. The results for storage root yield indicated that ‘Kalem’ genotype did not adopt well to the ecological conditions of the experimental sites, while other 3 genotypes adapted well to the environmental conditions of Tokat-Kazova.

Sweet potato is a sensitive plant to environmental variation. Several studies have revealed that changes in environmental conditions might have significant impact on its yield and quality [22–25]. Çalışkan et al. [49] reported that the storage root yield of 9 sweet potato genotype ranged between 21.81 and 112.60 ton ha^-1^ in Diyarbakır province, 19.36 and 85.60 ton ha^-1^ in Şanlıurfa province, 12.14 and 79.72 ton ha^-1^ in Adana province and 16.33 and 61.16 ton ha^-1^ in Hatay province. They concluded that sweet potato genotypes have successfully adapted to the South and Southeastern regions of Turkey. The storage root yield values obtained from ‘Havuç’, ‘Hatay Yerlisi’ and ‘Hatay Kırmızısı’ genotypes under Tokat conditions are similar to the yield values of the same genotypes reported by Çalışkan et al. [7]. Arslanoğlu and Hendekçi [44] obtained the highest storage root yield in Samsun coastal zone from ‘TP31’ genotype followed by ‘Hatay Red^’^.

### Dry matter and protein ratio of storage roots (%)

Dry matter and protein ratio of storage roots were significantly affected by different sweet potato genotypes (Table 5). Growing conditions in both locations had a significant effect on dry matter content of storage roots. Dry mater matter content in storage roots of sweet potato genotypes t Niksar location was higher than Kazova during both years of the study.

**Table 5:** Dry matter and protein ratio of different local sweet potato genotypes used in the current study.

Mean dry matter ratio (%)					Mean protein ratio (%)			
	Niksar		Kazova		Niksar		Kazova	
Genotype	2018	2019	2018	2019	2018	2019	2018	2019
Kalem	30.87 a	30.57 a	32.07^NS^	32.73^NS^	4.72 a	4.32 a	4.57 a	4.33 a
Hatay Kırmızısı	29.00 ab	27.97 ab	31.14	32.70	3.93 ab	3.75 ab	3.86 ab	3.67 ab
Hatay Yerlisi	31.02 a	30.37 a	33.36	34.93	3.53 ab	3.25 ab	3.57 ab	3.37 ab
Havuç	26.60 b	25.17 b	28.11	30.17	3.10 b	3.20 b	2.73 b	2.96 b
CV %	**4.89**	**5.57**	**4.80**	**4.27**	**21.73**	**19.92**	**23.69**	**21.20**
Mean	**29.37**	**28.52**	**31.17**	**32.63**	**3.82**	**3.63**	**3.68**	**3.58**

^NS =^ Non-significant, Means with different letters in a column are significantly different from each other at p<0.05.

The highest dry matter content of storage roots at Niksar during 2018 was recorded for ‘Hatay Yerlisi’ followed by ‘Kalem’, ‘Hatay Kırmızısı’ and ‘Havuç’ genotypes, respectively. During 2019, the highest dry matter content was recorded for ‘Kalem’ genotype. Oduro [50] and Gurmu [25] reported significant genotypic effect on dry matter content in sweet potato compared to environment and interaction effects. Significant effect of genotypes on dry matter content compared to location can be attributed to slight effect of environment on dry matter content among the sweet potato genotypes studied.

Dry matter content of sweet potato genotypes at Kazova location during 2018 ranged from 8.11% (‘Havuç’ genotype) to 33.36% (‘Hatay Yerlisi’). The highest dry matter content during second year of the experiment at Kazova location was obtained for ‘Hatay Yerlisi’ followed by ‘Kalem’, ‘Hatay Kırmızı’ and ‘Havuç’ genotypes, respectively.

The highest protein ratio of storage roots during both years at both locations was observed for ‘Kalem’ genotype, while the lowest for ‘Havuç’ genotype.

Carbohydrates, vitamins, minerals, and protein content of sweet potato were higher compared to the other root and tuber crops [51]. Yıldırım et al. [47] reported that mean protein content of 10 sweet potato genotypes ranged between 2.82 and 1.60%. The highest protein ratio was recorded for ‘Yang-Shu-1’ followed by ‘Hatay Kırmızısı’. The protein ratios reported by Yıldırım et al. [47] are similar or lower than the protein ratios reported in our study.

### Antioxidant activities

Antioxidant activities of genotypes at Niksar during 2018 were moderate compared to the standards. The antioxidant activity was not significantly different between sweet potato genotypes (Table 6). The highest antioxidant activity during 2019 at Niksar was recorded for ‘Kalem’ genotype. The highest DPPH scavenging effect during 2018 at Kazova was observed for ‘Kalem’ genotype. During 2019, the highest DPPH scavenging effect was recorded for ‘Hatay Yerlisi’ genotype. Teow et al. [10] reported a wide range of variation in antioxidant activity of 19 sweet potato genotypes.

**Table 6:** The DPPH scavenging activity of local sweet potato genotypes used in the current study.

	Niksar		Kazova	
Genotypes	2018	2019	2018	2019
**Kalem**	27.94 ± 2.13 c	40.48 ± 1.58 c	32.62 ± 2.48 c	29.44 ±0.78 c
**Hatay Kırmızısı**	25.19 ± 2.04 c	28.82 ± 1.32 d	28.66 ± 0.32 cd	31.96 ±2.33 c
**Hatay Yerlisi**	25.31 ± 1.31 c	36.63 ± 3.08 cd	32.35 ± 0.23 c	35.12 ±1.92 c
**Havuc**	27.24 ± 4.66 c	30.12 ± 2.73 d	26.80 ± 1.18 d	23.02 ±2.93 d
Standards				
BHT	60.54 ± 3.21 b		60.54 ± 3.21 b	
Trolox	94.10 ± 0.90 a		94.10 ± 0.90 a	

*Antioxidant results are reported as mean values ± SDs of three independent assays.

Values followed by the same letter are not significantly different (P>0.05).

### Conclusions

The genotypes significantly differed for yield and quality response and could adapt new climatic conditions as hypothesized. Temperature conditions in Kazova and Niksar were good enough for the sweet potato to complete the vegetation period. Temperature requirements of sweet potato plants in the experimental area could met 180 days from planting. The results of the study indicated that sweet potato can easily adapt to the climatic conditions of this region.
